# Mass Spectrometry-Based Methods for Identifying Oxidized Proteins in Disease: Advances and Challenges

**DOI:** 10.3390/biom5020378

**Published:** 2015-04-14

**Authors:** Ivan Verrastro, Sabah Pasha, Karina Tveen Jensen, Andrew R. Pitt, Corinne M. Spickett

**Affiliations:** School of Life and Health Sciences, Aston University, Aston Triangle, Birmingham, B4 7ET, UK; E-Mails: verrasti@aston.ac.uk (I.V.); pashas@aston.ac.uk (S.P.); k.tveen-jensen@aston.ac.uk (K.T.J.); a.r.pitt@aston.ac.uk (A.R.P.)

**Keywords:** oxidative post-translational modification, inflammation, cardiovascular disease, protein carbonyls, nitrotyrosine, chlorotyrosine, LC-MS/MS, precursor ion scanning, neutral loss scanning, multiple reaction monitoring

## Abstract

Many inflammatory diseases have an oxidative aetiology, which leads to oxidative damage to biomolecules, including proteins. It is now increasingly recognized that oxidative post-translational modifications (oxPTMs) of proteins affect cell signalling and behaviour, and can contribute to pathology. Moreover, oxidized proteins have potential as biomarkers for inflammatory diseases. Although many assays for generic protein oxidation and breakdown products of protein oxidation are available, only advanced tandem mass spectrometry approaches have the power to localize specific oxPTMs in identified proteins. While much work has been carried out using untargeted or discovery mass spectrometry approaches, identification of oxPTMs in disease has benefitted from the development of sophisticated targeted or semi-targeted scanning routines, combined with chemical labeling and enrichment approaches. Nevertheless, many potential pitfalls exist which can result in incorrect identifications. This review explains the limitations, advantages and challenges of all of these approaches to detecting oxidatively modified proteins, and provides an update on recent literature in which they have been used to detect and quantify protein oxidation in disease.

## 1. Introduction to Protein Oxidation

**Figure 1:**
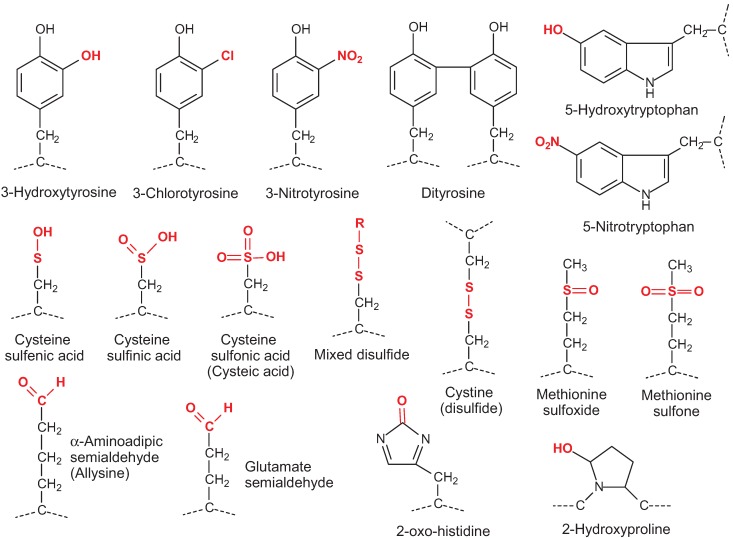
Structures of oxidized residues most commonly detected and studied by mass spectrometry. In mixed disulfides, R can be cysteine or glutathione (glutathionylation).

Many diseases have an oxidative aetiology resulting from activation of the immune system, mitochondrial dysfunction or environmentally-induced oxidative stress. Oxidative modification of proteins can have multiple effects, such as loss of enzymatic activity, functional alterations, loss of structural integrity, and protein aggregation [1]. Various different reactive and oxidizing species exist and vary in their reactivity to protein residues and sites. Metal-catalysed oxidation depends on the formation of hydroxyl radicals through Fenton chemistry; hydroxyl radicals are highly reactive and able to modify almost any site through hydrogen abstraction and peroxide formation, often leading to backbone fragmentation. The most susceptible side chains in proteins are the sulfur-containing cysteine and methionine side chains; the reactivity of cysteine with hydrogen peroxide depends on the pKa of the thiol group as the thiolate anion is a better nucleophile. Cysteine can also react with reactive nitrogen species to form nitrosothiols (Figure 1). Other residues that are commonly oxidized include histidine, proline, lysine and arginine, where hydroxylation or formation of aldehydes or ketones may occur. Reactive nitrogen compounds derived from peroxynitrite are often both nitrating and oxidizing. Sites susceptible to nitration include tyrosine (forming 3-nitrotyrosine) and tryptophan. Hypohalites can also react with aromatic residues to form halogenated products such as 3-chloro and 3-bromotyrosine [2].

Protein oxidation is often measured as a marker of oxidative damage and cellular stress, and a wide variety of methods exist, varying from simple global methods to specific approaches to detecting individual modified residues [3]. A commonly measured modification is carbonyl formation, which can occur on lysine, arginine, serine, threonine and proline residues following metal-catalysed oxidation or attack by hypochlorous acid. Carbonyl groups react with DNPH (2,4-dinitrophenylhydrazine) and other aldehyde reaction probes such as *N*'-amino-oxymethylcarbonylhydrazino-*D*-biotin, offering potential for colorimetric detection or selective enrichment approaches. Anti-DNP antibodies form the basis of carbonyl-focused western blotting (“oxy-blotting”) and ELISAs [3,4]. Total digestion followed by HPLC, LC-MS or LC-MS/MS has been used to investigate a wide range of oxidized amino acids [5], but these approaches do not provide information on the specific protein that has been modified, or the exact site of modification.

Mass spectrometry has been used for many years for identification and characterization of proteins, and is arguably the most informative method for determining oxidative modification of proteins currently available. This article gives an overview of advances and limitations of LC-MS/MS approaches for detecting specific non-enzymatic oxidative modifications to proteins, and summarizes their recent application in studies of disease.

## 2. Overview of Mass Spectrometry Methods for Protein Oxidation Analysis

**Figure 2:**
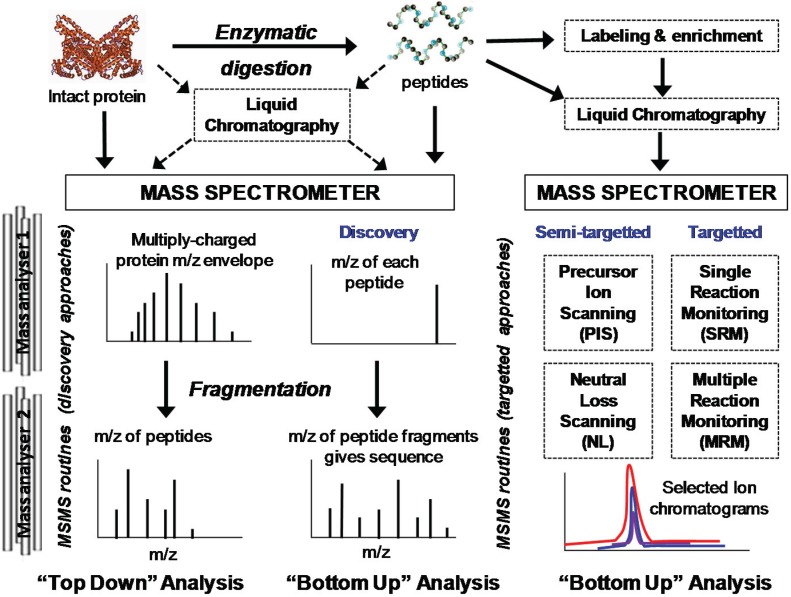
Summary of advanced methods for identification of proteins and oxPTMs. Labeling and enrichment can also be carried out at the protein level, but this approach is less common.

Mass spectrometry measures the mass-to-charge ratio (*m/z*) of ionized analytes, and as oxidative modifications alter the chemical composition of a protein, they change the *m/z* ratio of the intact protein and of the residues where the oxidation occurred; thus, MS is a powerful method for detecting oxidative post-translational modifications (oxPTMs) [2]. Mass spectrometry approaches for the analysis of proteins, both native or oxidized, have advanced substantially in recent years, and can essentially be divided into “top-down”, which involves analysis of intact proteins and their fragmentation within the mass spectrometer, and “bottom-up” analysis, in which proteins are enzymatically digested to a peptide mixture before being introduced to the instrument (Figure 2). The latter is by far the more common method, as it is extensively used in proteomics studies to sequence and identify proteins in biological samples, and has been extended to investigate protein oxidation. However, while identification of proteins using search engines to match experimental MS data against protein sequence databases is now routine, the analysis of post-translational modification, including oxidative modifications, continues to be extremely challenging. Consequently, there is a continual search for methodologies that facilitate identification of oxPTMs. This has led to the development of targeted mass spectrometry routines that search for peptides containing ions that are diagnostic for the presence of an oxidative modification, such as chlorotyrosine or methionine sulfoxide. Alternatively, the use of chemical reagents that react with oxidative modifications, which can be used as tags to label modified peptides or proteins, can facilitate both enrichment and detection and has seen significant recent development; carbonyl-reactive probes are a major focus of this approach. For all of these methods, an overarching aim is to be able to quantify the level of oxPTM, either in absolute terms or relative to the level of total protein. Advances in these different strategies are described in more detail in the following sections.

### 2.1. Sample Preparation and Digestion

An important practical consideration for any study of protein oxidation is how to minimize oxidative artefacts caused by sample processing. Bottom-up strategies usually involve digestion in solution or one or two-dimensional gel electrophoresis followed by in-gel digestion; both methods have been shown to introduce artefacts such as methionine, cysteine or tryptophan oxidation [6], so care is needed to minimise exposure to air and in the interpretation of results. Adventitious oxidation, such as artefactual *S*-thiolation of reactive, surface-exposed cysteine residues, has also been identified as a problem in top-down MS [7].

Protein digestion strategies for bottom-up approaches depend to a great extent on the type of experiment, but there are important considerations for mapping oxPTMs. Where comprehensive mapping of oxidative modifications of proteins is the aim and as close to complete sequence coverage as possible is required, or for studying modifications of specific residues within a protein where trypsin does not yield an appropriate peptide for MS, it is often necessary to use alternative proteases to the commonly used trypsin that cleave at different residues, or even combinations of proteases. Many alternative proteases with orthogonal activities to trypsin have been used successfully in recent years, including chymotrypsin (large hydrophobic), Asp-N (N-terminal to asp), Glu-C (N-terminal to Asp and Glu) and others [8,9,10]. Selective proteolytic cleavage may also be used to help to identify oxidative modifications; for example, AspN or GluC also cleave at cysteine sulfonic acid and trypsin at aminoethylcysteine [11], and pepsin can be used at low pH, which minimizes disulphide interchange [12]. New digestion methods that may help to minimize sample handling, and thus adventitious oxidation, include in-line digestion where the sample is passed through a column of beads coated with trypsin, which digests the proteins as they flow through [13,14]. MS friendly surfactant additives, such as ProteaseMax (Promega) [15,16] and Rapigest (Waters) [17], and on-membrane [18] or in-pellet digestion [15] have all been shown to improve sample digestion efficiency and recovery of peptides, and thus give increased sequence coverage and may improve coverage of modifications; in one study, 1000 *S*-glutathionylated sites on proteins were identified using in-pellet digestion [19].

### 2.2. Enrichment and Separation

In addition to limiting adventitious oxidation as mentioned in the previous section, it can also be useful to stabilize labile oxidative modifications that are genuinely sample-derived. Moreover, chemical labeling of modifications offers the possibility of enrichment by tag-specific binding systems, thus reducing the complexity of samples and facilitating detection of the oxPTM of interest. Affinity enrichment methods are useful for oxPTMs directly, using antibodies to the modification or chemical tag, or other resin-based capture agents, or based on chemical reactivity (for example thiopropyl sepharose [20]). However, significant care and accurate quantification, as well as appropriate experimental protocols, are necessary to minimise non-specific interactions with the solid support and identify these in the subsequent data analysis [21,22]. Immunoenrichment with antibodies against oxPTMs (for example anti-nitrotyrosine antibodies) has been used to enrich proteins from biological samples [23], although this is not always successful [24] and can introduce a high background of immunoglobulins and other proteins into the sample. In addition, the lack of specificity in immunoenrichment exhibited by many antibodies can significantly compromise this approach.

Overall there are many different chemical labeling and enrichment strategies for detecting and quantifying oxidations. The biotin-switch method has been developed to detect reversible cysteine oxidations such as disulfides, sulfenic acids (-SOH) and *S*-nitrosothiols (-SNOs) [25]. The principle is that free thiols are first blocked with an alkylating agent (e.g., iodoacetamide), then the oxidative modifications are selectively reduced; for example, using DTT for disulfides, ascorbate for SNOs or arsenite for sulfenic acids, followed by biotinylation with a thiol-reactive biotin reagent. This allows enrichment by avidin affinity capture [26]. Careful consideration also needs to be given to the protocols for these approaches to ensure residual reducing, oxidising or alkylating agents are properly quenched, or that a significant excess of reagent is used, at each step. The limitations of this approach are the low throughput and difficulty in localising the modification by MS, as ionisation efficiency and peptide fragmentation are often compromised by biotinylation. Recently, some of these issues have been resolved and a quantitative method developed using commercially available iodoacetyl tandem mass tag (iodoTMT) reagents, as described in Section 6 [27]. An alternative method developed for the enrichment of SNOs is the use of organic mercury columns, which involves covalent bond formation between the SNO and mercury. The modified proteins can be digested while bound on the column before elution and MS analysis [28]. Protein carbonyl groups are reactive and can also be labelled by nucleophilic reagents and linked to biotin for enrichment [29]. DNPH is a well-established carbonyl-label and has the advantage that it can act as the matrix for matrix-assisted laser desorption/ionisation (MALDI-) MS, which provides increased specificity and sensitivity for carbonyl-containing peptides and eliminates the need for upstream enrichment. DNPH-labelled peptides can also be analysed with data-dependent acquisition methods with ESI-MS [30]. This technique has recently been applied to a proteome-wide study of protein carbonyl groups generated by mild oxidation; 210 carbonylated proteins were identified with a total of 643 carbonyl locations detected in the HeLa cell proteome [31].

Chemical tagging approaches have also been used to detect the formation of protein bound nitrotyrosine; the initial step is reduction of the nitro group to an amine, which is more amenable to tagging. A wide variety of reduction, labeling and enrichment methods, for example using *N*-succinimidyl-*S*-acetylthioacetate [32] or dansyl chloride [33], have been reported, and reviewed recently [34]. Usually this approach is reported to give an improvement in selectivity, and indeed enrichment steps have often been regarded as essential. Several of the tags can also be used as reporters in subsequent MS analysis [33].

Developments in LC-separation are also desirable in order to reduce sample loading and improve separation of proteins and peptides. Gel eluted liquid fraction entrapment electrophoresis (GELFrEE) integrates gel electrophoresis separation within reverse phase LC, and eliminates the need for prior electrophoresis and sample processing before injection into the LC [35]. It has been applied to the detection of nitrotyrosine using the increased hydrophilicity of aminotyrosine (formed by reduction of nitrotyrosine with dithionite) and concomitant shift in chromatographic elution of modified peptides on reverse phase HPLC [36]. In contrast, for top-down studies the favoured method is Capillary Zonal Electrophoresis (CZE), which allows lower sample loading and has higher separation efficiency than reverse phase HPLC [37,38]. This can facilitate detection of oxidation in complex clinical samples, where protein concentration may be limited.

### 2.3. Intact Protein and Top-Down Analysis

Intact protein analysis, where MS is used to determine the mass of the intact protein and changes in mass can be indicative of modifications to the protein structure, is a well-established approach. Both MALDI and ESI have been used, although ESI is the more common method as it is generally able to give mass accuracies better than 1 in 10,000 on most instruments. This accuracy is due to the protein acquiring more than one charge during ionization, usually many different charges, giving rise to a number of peaks in the spectrum (since MS measures mass-to-charge ratio, each different charge state will give rise to a separate peak in the spectrum) [39]. This set of peaks can be used to help reduce errors in the calculation of the mass, and this has been enhanced further by the ability of high resolution instruments to separate the individual isotopic peaks for even very large proteins, enabling the analysis of larger proteins including antibodies [40] and even protein complexes [41]. However, this multiple charging means that only a limited number of proteins or different protein species can be present in the sample before signals start to overlap and deconvolution becomes more difficult. This method can provide useful information on the total load of modifications on an individual protein molecule [42], and has been applied successfully to detecting methionine oxidation [43], glutathionylated haemoglobin [44] and electrophilic modifications [45], although for small modifications, high-resolution instruments such as Q-TOFs, Orbitraps or FT-ICR MS are really needed for accurate determination of multiple different forms. However, in order to determine the site of modification, either bottom-up or top-down methods are needed. Top-down MS is an emerging platform that involves fragmentation of the intact protein within the mass spectrometer, and analysis of the large fragments produced. It requires high-resolution mass spectrometers and alternative fragmentation technologies that are not available on all instruments. It is currently limited in sensitivity and struggles to deal with complex samples, but has great potential for mapping protein oxidation [46,47,48]. Most top-down studies have been conducted *in vitro* with low molecular weight proteins, although more recently a range of 30–80 kDa proteins in a whole cell lysate of *P. aeruginosa* have been analysed [38]. The top-down approach has the advantage of providing additional information on the relative occupancy of oxidation and relationships of oxidised residues to one another in the whole protein [46,49,50]. For example, methionine oxidation and nitrotyrosine have been detected and quantified in calmodulin following incubation with lipopolysaccharide (LPS)-activated macrophage lysate [50]. The oxidation of multiple methionine residues has also been quantified using top-down approaches in filgrastim, a granulocyte colony-stimulating factor, to determine the effects of methionine oxidation on biopharmaceutical shelf life [49]. However, despite these reports, the methodology is still some way short of being applied to the detection of protein oxidation in disease.

### 2.4. Bottom-Up Analysis

Bottom-up proteomics differs from top-down analysis in that the proteins are digested to peptides as mentioned in Section 2.1. Specific labeling and enrichment strategies can be implemented at this stage, as described in the previous section, although label-free methods are more common in standard proteomics. In all bottom-up methods, quantification is restricted to the peptide level, and cannot be used to infer relationships between oxidations on different peptides within an individual protein.

Bottom-up protein analysis is most commonly conducted by LC-MS/MS using either untargeted analysis (often referred to as a shotgun or discovery approach, and described further in Section 3), or semi-targeted/targeted approaches [51], which are described in Section 4. The former is most common, but the limitations of this approach for detecting oxidative modifications lie in the automated selection of the peptides to be fragmented, which tends to be those that give strongest signals in the preliminary MS scan, whereas oxidized peptides are typically present at very low abundance [52]. Hence there has been significant effort recently in developing targeted and semi-targeted methods that depend on scanning for reporter ions in the MS or MS/MS spectra that are diagnostic for the presence of an oxidative modification. This requires some prior knowledge, at least of the fragmentation characteristics of the oxidative modification, and for some methods additionally the specific peptide modified.

These mass spectrometry-based methods can be implemented either in label-free or label-dependent strategies. Label-free approaches are widely used in standard proteomic analysis, and even for analysis of oxidatively modified proteins they have the advantage of less sample manipulation. With regard to identification of oxidative modifications, label-dependent methods usually refer to modification-specific chemical tagging, in contrast to the isotope-labeling techniques used for more generic quantification, although a few studies have combined these methods for quantifying modifications (Section 5). Label-dependent MS approaches often take advantage of reporter ions from the label to indicate the presence of a modification in a peptide, which can then be targeted for further analysis, and are discussed further in Section 4.

## 3. Untargeted Mass Spectrometry and “Discovery” Approaches

A large proportion of proteomics and MS methods are focused on identification and quantification of specific proteins in diverse samples, in order to understand proteomic changes in disease or other conditions. Analysis of oxidative modifications in proteins represents a much smaller field, and although specific methodologies are being developed and utilized, much research is still carried out using untargeted approaches (Figure 2).

### 3.1. Use of Search Engines for MS Data and Analysis of oxPTMs

LC-MS/MS experiments generate very large datasets that are difficult to manually analyse, and consequently many statistical search engines have been recently introduced or further developed for identification of proteins from MS/MS data; some of the most commonly used examples are Mascot, PEAKS, Sequest, ProteinPilot, Tandem, Ommsa and Phenyx [53,54,55,56] (Table 1). While generally these programmes work very well for identifying proteins, more issues arise when trying to identify oxPTMs, at least using standard parameter settings [57], and some of the advantages and disadvantages that have been observed are identified in Table 1.

**Table 1:** Comparison of the advantages and disadvantages of the most commonly used search engines for peptide and protein identification.

Search Engine	Method	Advantages	Disadvantages
**Mascot**	Uses a probability modelling algorithm and protein database searching. Matches experimental peptide and fragment ion masses to ones generated *in silico* from databases.	User-friendly interface. Provides an error-tolerant search facility. Sophisticated but complex data export possibilities.	Very reliant on user input for correct identification of oxPTMs, otherwise false positives and negatives occur.
**Sequest**	Uses an algorithm based on a cross correlation function, plus protein data base searching. Matches experimental peptide and fragment ion masses to ones generated *in silico* from databases.	User-friendly interface. Provides an error-tolerant search facility.	Very reliant on user input for correct identification of oxPTMs, otherwise false positives and negatives occur.
**ProteinPilot**	Uses a sequence tag method plus protein database searching.	User-friendly interface. Potentially better at identifying unsuspected modifications.	If the initial sequence tag is incorrectly identified, the experimental peptide will not be matched to the correct peptide. Long analysis run times.
**pMatch**	Spectral library searching against experimentally-derived data.	Has been reported to be better at identifying PTMs, and specifically at coping with the unusual fragmentation of peptides caused by PTMs.	Since this method uses a spectral library, the peptide will only be identified if the spectra are available in the spectral library.
**MS Amanda**	Based on a binomial distribution function. Protein data base searching. Matches experimental peptide and fragment ion masses to ones generated *in silico* from databases.	Reported to be better at identifying peptides of higher *m/z* than Mascot and Sequest.	Very reliant on user input for correct identification of oxPTMs, otherwise false positives and negatives occur.

Many search engines offer the possibility of including a wide variety of oxPTMs as variable modifications [58], but the number of modifications that can be searched in parallel is usually limited to 3–4 to minimize false positive identifications [59], which can be limiting when heterogeneous oxidation has occurred. oxPTMs also add additional complications to searching data. For example, the spectrum of a peptide containing methionine sulfoxide will include a neutral loss of 64 Da (-CH_3_SOH), which complicates interpretation of the spectrum and sequencing, although this can be improved using alternative fragmentation methods [60]. Protein Pilot works in a different way to Mascot, Sequest and MS Amanda [61], and is less affected by these constraints, so may have advantages for the analysis of complex oxPTMs. It is based on the Paragon algorithm and uses small sequence tags generated by *de novo* sequence analysis of parts of the experimental data. The sequence tag is searched against a protein database and any sequence in the database that matches the sequence tag is investigated for a fit to the experimental data set, in an iterative approach. All possible PTMs are allowed for in the error-tolerant mode. This has the advantage of being a non-statistical method in which a sequence can be constructed with the inclusion of a wide variety of oxPTMs, but if the initial sequence tag was incorrectly identified, the final peptide will also be incorrect, and the large search space results in a time-consuming process. Mascot also incorporates an error-tolerant search function that has been substantially developed in recent releases, and does not require a list of anticipated modifications; in this mode all possible PTMs are tested against the theoretical peptide and fragment ion masses, and the PMT that gives the best match to the experimental data is reported as a match. Again, this increases the search time and tends also to increase the false positive rate.

A new search engine, MS Amanda, has been specifically designed for high resolution instruments; it has a different scoring function for identification of the peptides and has been reported to identify many more peptides than both Mascot and Sequest, including ones with multiple modifications [62]. The resulting increase in sequence coverage could help in identification of oxPTMs, although the algorithm is still limited by the same issues. An entirely different approach to identifying peptides that is gaining popularity and may help to overcome the limitations of searching for oxPTMs involves spectral library searching using a search tool such as pMatch [63]. This method compares the experimental MS data with previously acquired spectra in a spectral library using an open search. This allows the search engine to search for unknown and unspecified modifications, but depends on them being present and correctly identified in the library.

While statistical software is often used to detect oxidative modifications, comparisons of the results from different search engines are rarely performed. Dorfer *et al.* compared the ability of several search engines to identify proteins and post translation modifications [62], while Moskovitz used three search engines to detect and localize methionine oxidations [64]. In both instances validation by manual *de novo* sequencing was not performed; this makes it difficult to determine which search engine is the most reliable for determining the presence and localisation of oxidative modifications.

### 3.2. The Importance of Data Validation

**Figure 3:**
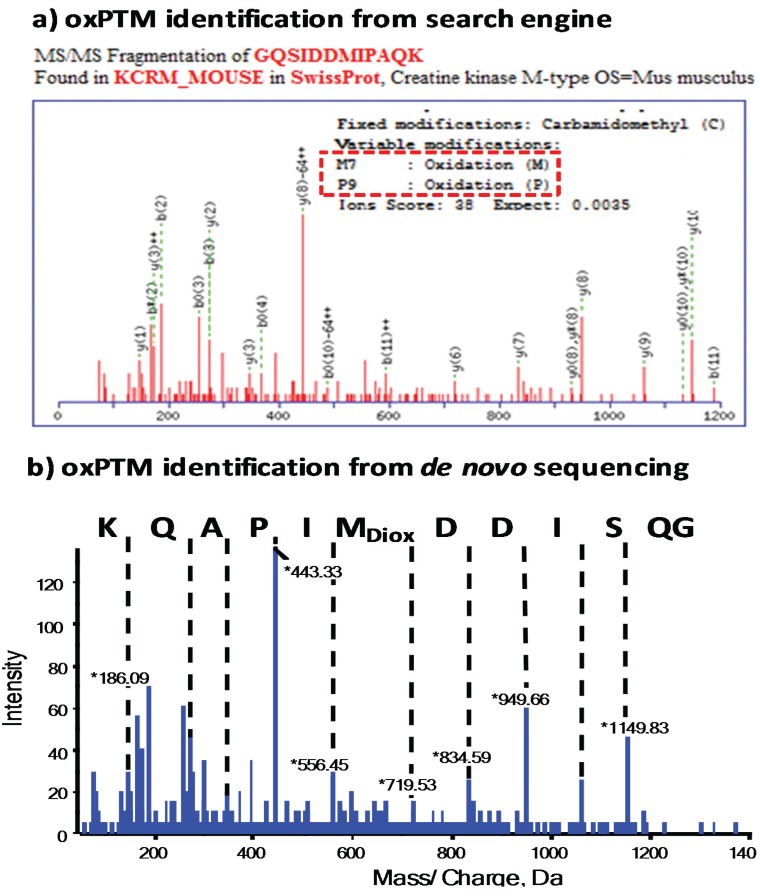
Incorrectly assigned oxidation to proline using a probability-based search engine. (**a**) Search engine identified 2 modifications on one peptide: methionine-7 mono-oxidation and proline-9 oxidation; (**b**) *de novo* sequencing showed that methionine is dioxidised.

In view of the potential pitfalls described above, it is essential to validate the MS/MS data by *de novo* sequencing to demonstrate both the presence and location of the modification in the sequence (Figure 3), despite it being time-consuming. This process has been reviewed previously [65] and guides to support different levels of expertise are available, e.g., [66]. The software packages to facilitate *de novo* sequencing are continually being developed, such as computer aided manual validation software (CAMV), which is compatible with iTRAQ-labeling quantification experiments and has been reported to remove approximately 10% of false positives [67]. Other software packages that aid in manual validation include PepNovo^+^, PEAKS, pNovo, MS-GFDB. UniNovo is reported to be best for manual validation of Orbitrap MS data [68]. Open source software to improve the user interface of packages such as PepNovo^+^ is also available [69]. A new approach to *de novo* sequencing by combining data from bottom-up and top-down MS approaches has recently been reported to achieve high sequence coverage and accuracy [70]. These tools are important, as more widespread use of *de novo* sequencing to validate oxPTMs is needed to ensure that correct relationships between oxPTMs and disease are being deciphered.

## 4. Reporter Ion-Based Methodologies

The term reporter ion refers to the formation of ions that are diagnostic for the specific analyte or type of analyte of interest, usually product ions from the fragmentation of the peptide. Reporter ions have been used in label-free analysis where a sufficiently unique fragment of an oxidized residue has been identified; alternatively, several oxidation-specific chemical probes that are MS compatible and give diagnostic fragmentations have also been developed. In semi-targeted methods, the fragmentation products are diagnostic but the precursors are unknown (Section 4.1 and Section 4.2), whereas in fully targeted routines both the precursor ion and fragment ions are used as reporters (Section 4.3). In all of these approaches, specificity for oxPTMs is improved compared to untargeted analysis and relative quantification can be achieved using the relevant precursor ions for oxidized and native peptides.

### 4.1. Semi-Targeted MS/MS Analysis

Neutral loss scanning (NL) and precursor ion scanning (PIS) are two MS/MS routines that enable classes of molecular ions to be identified based on a structural feature with a characteristic fragmentation pattern. In precursor ion scanning the second analyser is fixed to detect a specific fragment ion and scans for precursor ions that generate this product ion upon fragmentation. For analysis of oxidized peptides, immonium ions from oxidized residues have been the most commonly reported reporter. For example, there have been several reports of the use of the nitrotyosine immonium ion at *m*/*z* 181.1 [71], and chlorotyrosine (*m*/*z* 170.1), hydroxytyrosine (*m*/*z* 152.1 Da), and hydroxytryptophan (*m*/*z* 175.1 Da) have also been tested [42]. However, for each of these isobaric ions from fragmentation of non-modified peptides, false positives have been reported; this is particularly a problem on low-resolution instruments where the interfering ions are not resolved from the target fragment ion [52,59,72]. One approach developed to overcome this problem is a further fragmentation step to yield a more unique combination of MS/MS and MS^3^ (MS/MS/MS, a further fragmentation of a selected ion generated in the MS/MS analysis) diagnostic ions; this has been reported for chlorotyrosine, nitrotyrosine, hydroxytyrosine and hydroxytryptophan in model proteins and cell lysates using an indirect scanning routine [42,73]. Greater specificity can also be obtained using higher-resolution instruments, as has been reported for nitrotyrosine [73,74].

In neutral loss scanning, the diagnostic fragment is uncharged and is monitored by scanning in both analysers with a mass offset corresponding to the mass of the fragment. This methodology has been used to identify the presence of oxidized methionine, which has a characteristic neutral loss of 64 Da (corresponding to methanesulfenic acid, CH_3_SOH) [75], and has been applied to measure in oxidation of calmodulin [76]. Oxidized cysteine residues fragment in a similar way with different neutral losses depending on the extent of modification, as reviewed recently [2]. Schiff base and Michael adducts of 4-hydroxynonenal with nucleophilic residues can also be monitored by neutral losses of 138 Da and 156 Da respectively, and has been demonstrated in plasma proteins [77].

In addition to these label-free semi-targeted methods, label-dependent approaches have been reported. For example, carbonyl-containing proteins or peptides can be labeled with DNPH to form hydrazone adducts, which can be analysed in negative ion mode based on a precursor-like scan for diagnostic fragments at *m/z* 152.0, 163.1 and 179.0 [30]. One of the advantages of this technique is the elimination of the need for upstream enrichment during sample preparation, as demonstrated in its application to analysis of oxidized proteins in bovine serum albumin and extracts of HeLa cells [30].

### 4.2. Narrow-Window Extracted Ion Chromatograms

An alternative strategy that has been developed recently as a result of the increasing availability of high-resolution mass spectrometers involves generating extracted ion chromatograms (XICs) of accurate mass reporter ions from MS/MS data [73]. One study used this technique to quantify the levels of *S*-glutathionylation in haemoglobin F subunits, as evidence of oxidative stress in premature infants [44]. However, this strategy has limitations in complex samples where the likelihood of isobaric peptides is higher. Alternatively, XICs of diagnostic product ions can be used to mine data for oxidative modifications. Recently, this approach was utilized for reporter fragments of nitrotyrosine, chlorotyrosine, allysine and for adducts of oxidised phospholipids with proteins [73]. The use of a very narrow mass window (0.05 Da) extracted ion chromatogram allowed exclusion of many false positive signals from isobaric ions. An advantage of this method is that existing data can be mined retrospectively for other modifications, as long as a unique reporter ion can be identified, but a disadvantage is that it involves significant manual processing [59,73].

### 4.3. Targeted Methods of Analysis

Fully-targeted MS/MS approaches involve two related techniques: single reaction monitoring (SRM) and multiple reaction monitoring (MRM), where both the precursor ion and product ion masses are fixed for the analyte of interest [78]. OxMRM, which combines MRM with protein purification and labeling of oxidised cysteine residues with isotope labeled *N*-ethylmaleimide, has been reported to improve sensitivity [79]. Although these targeted approaches are not a discovery strategy as prior knowledge of analytes is required, they represent the most accurate available MS-based quantification tool and can be conveniently used in hypothesis-driven studies upon optimization of chromatographic and mass spectrometric features; further developments of the rapidly developing PeptideAtlas to include modifications may greatly extend their utility [80].

## 5. Quantification of (ox)PTMs

In order to obtain meaningful data on protein oxidation in biological or clinical samples, it is crucial to be able to obtain accurate quantitative information about the oxPTMs and their relative abundance both within and between samples. Quantitative proteomics strategies can be generally divided into label-free and label-based approaches. Label-free techniques rely on comparisons of the abundance of the analyte ion intensities directly, with appropriate normalization, whereas label-based approaches rely on metabolic or chemical labelling of samples with differentially stable isotope labelled reagents and comparison of the ion intensities from these.

### 5.1. Label-Free Methods of Quantification

Label-free methods are becoming the most popular for relative quantification, as they are relatively easy to implement and a number of free, open source software packages are available for analysis. However, label-free strategies also need to be used with care when analysing oxPTMs, as these modifications will affect both peptide ionisation efficiency and MS/MS fragmentation pattern, complicating any comparative analysis. Hence, great care needs to be taken in comparing ion intensities between any given peptide and its modified form, especially when the modification removes (e.g., lysine to α-aminoadipic semialdehyde) or introduces (e.g., cysteine to cysteine sulfonic acid) ionizable groups, or alters polar residue composition; using this approach to determine a percentage modification can only be semi-quantitative at best, as changes can be very marked. For example, we recently reported a 2.6 fold increase in relative signal intensity on nitration of a peptide [81]. Using the loss of the native peptide ion intensity could be an alternative, but only where there is significant modification, as quantification accuracy is rarely better than 10%. The use of tags that improve ionization, for example the iTRAQ (isobaric tags for relative and absolute quantification) label discussed below, may help to improve this, although relative quantification of the same modified peptide between samples generated under different conditions, or using absolute quantification with a labeled peptide such as in the “protein-AQUA” strategy [82] also discussed below, are the only reliable methods.

The two fundamental strategies currently used in label free quantification are spectral counting and feature-based quantification. The different methods have been reviewed elsewhere [83]. Methods based on spectral counting rely on the number of identified MS/MS spectra corresponding to a given protein as a measure of protein relative abundance. While spectral counting has been used effectively in investigations of protein expression changes, including those induced by oxidative stress [84,85], it is focused on protein-level quantification and is not well suited for the specific analysis of oxPTMs (or many other PTMs) due to their often relatively low stoichiometry and abundance. Feature-based quantification methods rely on the comparison of summed peak intensities for each peptide in each LC-MS run, following software alignment the different LC-MS runs so that the same features are aligned in each data set. With the increasing interest in label-free methods, a new generation of software solutions capable of processing large amount of high resolution data have recently become available, including Progenesis QI (Non Linear Dynamics, Newcastle upon Tyne, UK), msInspect/AMT [86], MAxQuant [87], Rosetta Elucidator (Rosetta Biosoftware, Seattle, WA, USA), OpenMS [88] and Superhirn [89]. Although the use of label-free analysis for biomarker discovery in biological samples has been reported [90], few studies have reported the use of label-free software based methods for the quantitative determination of specific oxPTMs. As for spectral counting, a limitation has been that the methods are generally focused on protein-level quantification, and the identification and quantification of individual PTMs has been challenging; however, this is improving, and the latest versions of many of the programmes now incorporate specific methods for highlighting PTMs. In one recent study, reversibly oxidized cysteines in the membrane proteins of human erythrocytes have been quantified using a robust computational software-based approach and validated by matching the modified peptides against Protein Data Bank entries [91].

### 5.2. Label-Dependent Methods of Quantification

Label-dependent methods rely on the incorporation of isotope labels into the peptides prior to mass spectrometry analysis. Isotope labels can be introduced at various stages of the experimental workflow, depending on type of sample and MS approach. The following section will concentrate on methods specific for oxPTMs.

The use of chemical or enzymatic methods to incorporate the isotopic label after protein digestion has been implemented effectively in a wide range of studies to detect and quantify oxPTMs. One of the first was ICAT (isotope coded affinity tags), and its cleavable version cICAT, which are commercially available cysteine-specific tags based on an iodoacetamide (IAM)-based thiol-reactive group, and also carry an affinity tag for the enrichment of tagged peptides. ICAT has been effectively used to quantify evidence of cysteine oxidation in complex protein mixtures [92,93]. It has the advantage that enrichment can improve the depth of the analysis, but a significant disadvantage is that the presence of the ICAT tag can affect the quality of MS/MS data [94]. Other reagents have been recently developed for cysteine oxidation analysis. Isotope-labeled *N*-ethylmaleimide (NEM) has been used in a targeted MS approach to monitor the redox status of reversibly oxidized cysteines and the detection and analysis of cysteine disulfide bonds [95], and IAM based strategies are now being further developed for the detection and quantification of protein *S-*nitrosothiols (SNOs, recently reviewed in [92]). The recently commercially available iodoacetyl tandem mass tag (iodoTMT) six-plex reagent has been used for MS identification and quantification of SNOs [27,96], as well as other cysteine oxidations such as glutathionylation, nitrosoglutathione, and disulfides [96]. The TMT isobaric tags have been adapted recently for the comparison of the relative abundance or cysteine site occupancy by SNOs and sulfenic acids [97], and exploited for the detection of SNOs in LPS-stimulated microglial cells [27].

The use of iTRAQ, which labels primary amino groups and was developed for general quantification studies, has been extended for analysis of oxPTMs [19]. In combination with NEM-based thiol-blockade, iTRAQ has been used to identify the redox-sensitive reversibly-oxidized cysteines in proteins and to quantitatively assess the oxidation states of individual cysteine residues [98]. iTRAQ has recently been modified to detect other oxPTMs including protein carbonylation [99] and to selectively label and quantify 3-nitrotyrosine, both alone and in combination with precursor isotopic labeling [100]. Promising results in the detection of other oxPTMs have also been generated using specific enzymatic reactions to place the isotope tag at specific amino acid groups. For example, enzyme-catalysed O^18^-based labeling has been successfully used for accurate quantification of oxidized methionine [101].

An extension of label-dependent methods is absolute rather than relative quantification, which can be particularly valuable for clinical biomarker analysis. The most commonly used method is AQUA [82], where a stable isotope-labeled version of the peptide of interest is synthesized and used as an internal standard, but this has not yet been applied to oxPTMs. iTRAQ-labeled internal standards have also been recently used in combination with targeted MS approaches to quantify evidence of proteolytic post translational modifications such as proteolytic cleavage [102] or phosphorylation, but again this method has yet to be applied in oxPTM analysis.

## 6. Applications *in Vivo* and in Disease

OxPTMs can be classified either as reversible modifications, most commonly the lower oxo-forms of cysteine and methionine, or irreversible modifications, including cysteine sulfonic acid, methionine sulfone, and most oxidation products of other residues. The reversible oxPTMs have generated much interest, as evidence is emerging for their role in redox signaling [2]. An increasing number of proteins have been found to be regulated by reversible oxidation of cysteine to sulfenate and disulfide forms [103,104], and this has been shown to contribute to physiological control of signaling pathways governing cell fate, such as apoptosis, proliferation or inflammatory processes [105]. Some of the best known examples include protein tyrosine phosphatases such as PTP1B, apoptosis signal-regulating kinase (ASK-1), caspases and peroxiredoxin [106,107]. Other more recently discovered redox-regulated proteins include the nuclear signalling protein HMGB1 [108] and Hsp33 [109]. These enzymes contain thiolates that are particularly susceptible to oxidation by hydrogen peroxide, which can be generated for example by NADPH oxidases following activation of growth factor or other receptors. The role of SNOs in enzyme regulation and signalling is also gaining recognition [110], as in studies on mitochondrial complex I [111]. Interestingly, there is growing support for the concept that tyrosine nitration has a role to play in protein redox signaling [112,113]. While often these are normal, physiological processes, there is also evidence that they can be dysregulated in disease or aging, and there have been some excellent reviews on this topic recently [103,114], including the application of mass spectrometry to support these studies [113,115]. Consequently, the following sections focus instead on examples of stable and irreversible modifications to proteins in specific diseases and their potential as biomarkers.

### 6.1. Considerations for Clinical Sample Type in oxPTM Analysis

Despite advances in technology, the determination of oxPTMs in biological and clinical samples remains challenging owing to sample complexity, low abundance of the modifications, and potential for adventitious oxidation [116]. The low abundance of modifications often encountered *in vivo* means that many studies are initiated by *in vitro* analysis of highly modified proteins. These often bear little relationship to the low levels of oxidative modification encountered in clinical samples (e.g., nitration [117]), which means they are relatively poor models for physiological protein modification. This is compounded by the poor quantification of some methods, for example in carbonyl and glycation analysis [116,118,119].

The type and abundance of oxPTMs is dependent on the sample type. The main sources of clinical samples for proteomics are body fluids and tissue extracts. Urine and blood are by far the most widely studied fluids, owing to the relative ease of their acquisition. Although urine can be obtained non-invasively in large volumes and is known to contain a more than 1500 different proteins [120], their concentration is too low for routine detection of oxPTMs. Consequently, there have been more studies of free oxidized amino acids as markers of protein oxidation. Additionally, collection urine is more susceptible to adventitious oxidation during the excretory process. Another non-invasive biological material is exhaled breath condensate, which contains a variety of proteins and has potential for early diagnosis of lung cancer [121]. In asthma patients, exhaled breath condensate has been found by targeted mass spectrometry-based methods to contain free 3-nitrotyrosine [122,123]. Plasma is a better source of concentrated proteins (more than 490 proteins have been resolved [124]), and abundant plasma proteins such as albumin [125] and fibrinogen [126] are often investigated. Protein analysis can be achieved using very small volumes of blood, for example from pinpricks, especially if combined with novel approaches such as paper-spray mass spectrometry [127], although this has not as yet been applied to oxPTMs.

A limitation of plasma is that it reports on the systemic status rather than being disease or organ-specific; consequently, it can be desirable to study protein oxidation in other body fluids. For example, cerebrospinal fluids have been used to detect products of protein oxidation in Alzheimer’s disease patients [128,129], and synovial fluids have been used for the detection of free and protein-bound 3 nitrotyrosine in osteoarthritis [130]. Protein oxidation has also been detected in saliva, seminal fluid, and amniotic fluid [131,132,133]. Ultimately, information about protein damage in organs requires the use of tissue biopsies to assess the local level of oxidation. Mass spectrometry-based procedures have been used on tissue biopsies of tumours [134] and virus-infected tissues [135], and oxidized proteins have been reported in surgical biopsies of diseased human tissues such as heart [136] and brain [137] tissue. Even with the small sample amounts obtained by needle biopsies, modern approaches and high-resolution instruments can profile proteins [138]. MALDI imaging has recently been used for proteomic analysis of needle-core biopsied human pancreatic tumour tissue spotted on microarrays, and evidence of protein oxidation was reported [139].

### 6.2. MS Analysis of Protein Oxidation in Disease

A major driver for analysing protein oxidation in biological or human samples is to determine their importance in disease [140]. This has two potential benefits: an improved understanding of their role or mechanism in the pathological condition, and the identification of improved biomarkers for diagnosis. Especially for development of clinical biomarkers, much research has been done on the analysis of oxidatively modified amino acids, such free nitrotyrosine or chlorotyrosine, oxidized tryptophan products, advanced glycation endproducts (AGEs), lipoxidation adducts, and thiol-containing compounds, and many well-established targeted MS methods are available [141,142,143,144,145,146,147,148,149]. Although these methods are very useful for gaining an overview of global oxidative damage, they do not yield information on the target proteins that have been modified or localize the modification on the protein. The desire for greater mechanistic insight has led to the development of the MS methods described in the previous sections, and in recent years the application of both label-free and label-dependent mass spectrometry methods to clinical analysis has grown exponentially.

This section will summarize important findings and provide an update on the analysis of oxidized proteins in disease. In some studies, elevated levels of oxidized proteins and oxPTMs in disease were observed, suggesting their potential as biomarkers, and these findings are summarized in Table 2. Although protein identification data for the protein-bound oxPTM(s) detected is provided in all these articles, it is important to note that not all of them report site-specific information about the modifications, and this limits the confidence of the oxPTM analysis. The oxPTMs that have most often been associated with human disease onset are protein carbonyls, 3-nitrotyrosine, 3-chlorotyrosine, dityrosine, cysteic acid, cysteine disulfide bonds, cysteine *S*-glutathionylation, cysteine *S*-nitrosylation, methionine sulfoxide and methionine sulfone.

**Table 2:** Summary of recent studies where increased levels of oxPTMs in disease have been detected using MS techniques.

Modification Type	Disease	Method	Sample Type	Protein Type	Oxidation Sites Identified?	Reference
Carbonylation	Alzheimer’s disease	DNPH, MALDI-TOF/MS	Blood (human)	Fibrinogen γ-chain precursor protein, α-1-Antitrypsin precursor	no	Choi *et al.*, 2002 [150]
Carbonylation	Aging	Avidin affinity, LC-MS/MS	Brain tissue (mouse)	Brain proteins	yes	Soreghan *et al.*, 2003 [151]
Carbonylation	Aging	FTCl-labeling; 2DE-MS	Liver tissue (mouse)	Cytosolic liver proteins	no	Chaudhuri *et al.*, 2006 [152]
Carbonylation	Aging	ITRAQ/LC-MS/MS	Skeletal muscle (rat)	Mitochondrial muscle proteins	no	Feng *et al.*, 2008 [153]
Carbonylation	Mild Cognitive impairment and Early Alzheimer’s disease	DNPH, MALDI-TOF/MS	inferior parietal lobule (human)	CA II, Syntaxin binding protein I, Hsp70, MAPK kinase I, FBA-C, PM-1, GFAP	no	Sultana *et al.*, 2010 [154]
Carbonylation	Aging	ARP-labeling, MS/MS	Heart (rat)	Cardiac mitochondrial proteins	yes	Chavez *et al.*, 2011 [155]
Carbonylation	Diabetes	ITRAQ/LS-MS/MS(SRM)	Plasma (rat)	Plasma proteins	yes	Madian *et al.*, 2011 [156]
Carbonylation	Obesity-induced diabetes mellitus	ARP-labeling RPC-MS/MS	Plasma (human)	Plasma proteins	yes	Bollineni *et al.*, 2014 [157]
Carbonylation	Breast cancer	iTRAQ	Plasma (human)	Plasma proteins	yes	Madian & Regnier, 2010 [29]
Carbonylation	Ischemia/reperfusion	2D-PAGE-MALDI-TOF/TOF/MS/MS,	Hippocampus (monkey)	Hsp70-1, DRP2 isoform 2, GFAP, β-actin	yes	Oikawa *et al.*, 2009 [158]
Carbonylation, cysteic acid, MetO, MetO_2_	Alzheimer’s disease, Parkinson’s disease	2D-PAGE, MALDI-TOF/MS MALDI-TOF/TOF/MS/MS, HPLC-ESI/MS/MS MALDI-MS/MS	Brain (human)	DJ-1	yes	Choi *et al.*, 2006 [137]
3-NO_2_Y	Cancer	NTAC-based MALDI–LTQ MS/MS	Non-functional pituitary adenoma tissue (human)	NTAC-enriched proteins	yes	Zhan & Desiderio, 2006 [120]
3-NO_2_Y, 3-Cl-Y	Influenza	LC-MS/MS	Serum (mouse)	Serum proteins	yes	Kumar *et al.*, 2014 [159]

Protein carbonyl formation is one of the most studied and well-established markers of oxidative stress-related human diseases [160]; usually chemical tagging for enrichment is used, as described in Section 2.2. Many clinical and disease-related investigations used untargeted MS or MS/MS methods to analyse gel spots from 2D-electrophoresis of DNPH-derivatised proteins techniques; this identifies the proteins present in gel spots that have been identified as carbonyl-containing by immuno-staining, but it is important to remember that unless the modification has been localized on the proteins of interest by MS/MS analysis and ideally by *de novo* sequencing, the identification of carbonyl-modified proteins is tentative. Using such approaches, evidence of increased levels of protein carbonyls have been detected in tissues from patients Alzheimer’s disease [132,150,154,161], and in aged rat skeletal muscle with quantification by iTRAQ based-methods [153].

In other studies, the DNPH label or other chemical tag has been further utilized for targeted MS/MS analysis. For example, protein carbonylation sites have been determined and validated in rat cardiac mitochondrial proteins using aldehyde/keto reactive probes (ARP) and avidin-based affinity enrichment coupled with LC-MS/MS [155]. The methodology was subsequently applied to study adducts of reactive lipid aldehydes in hearts of young and old rats, and interestingly the level of hydroxyhexanal-modified proteins was higher in mitochondria from young animals, in line with the concept that these mitochondria contain higher levels of omega-3 (*n*−3) fatty acids. On the other hand, the location and increased levels of carbonyls have been reported in proteins of aged mouse brain [151]. Ischaemia/reperfusion is known to cause oxidative stress, and increased carbonyl modification of Hsp70 and several neuron-specific proteins have been observed in monkey hippocampus [158]. Bollineni *et al.* used the carbonyl-reactive probe *O*-(biotinylcarbazoylmethyl)hydroxylamine followed by avidin affinity chromatography to demonstrate differences in the profiles of carbonyl-containing proteins in plasma of obese subjects and patients with type 2 diabetes [157]. The carbonyl status of 35 different proteins has also been mapped in diabetic rat plasma, and was found to increase significantly in 11 of them [156]. This group also investigated carbonyl-containing proteins in plasma of breast cancer patients and found that they were strongly associated with the breast cancer type-1 susceptibility protein Brca1 [162]. These studies built on a high through-put methodology incorporating carbonyl-labeling and iTRAQ for quantifying protein carbonyl analysis in human plasma [29].

The redox processes of cysteine, both reversible and irreversible, are also of emerging clinical relevance [163,164]. Reversible cysteine oxidation has been found using proteomics approaches in the skeletal muscle of aged rats [165]. Untargeted MS/MS approaches have been used to provide evidence of irreversible cysteine oxidation in different proteins in brain tissue of patients with Alzheimer’s or Parkinson’s diseases [137,161,166]. Cysteine SNOs have been also linked to aging and Alzheimer’s disease, according to a proteomics study on human brain samples [167]. Interestingly, cysteine SNO formation has been reported in mouse models of ischaemia/reperfusion injury using SNO-RAC (*S*-nitrosothiols resin affinity capture) in combination with label-free based quantification [168]. Ischemia/reperfusion was also found to cause reversible oxidation of cysteine in heart tissue of mice using Redox-ICAT for quantification by MS/MS [169]. A similar approach has been used to study redox switches in liver mitochondrial protein samples during cadmium toxicity in rats [170].

Methionine oxidation has been much less studied than cysteine oxidation, but evidence is emerging for links to a number of human pathologies, including Alzheimer’s [171] and Parkinson’s diseases [172]. Protein-bound methionine sulfoxide (MetO) was found to be elevated in the plasma of diabetic patients [173] as well as in the brain tissues of patients affected by Alzheimer’s and Parkinson’s disease.

Oxidatively modified tyrosines have also been proposed as consistent biomarker of several inflammatory and chronic human pathologies [174]. One of the most studied markers of peroxynitrite-mediated damage in MS-based studies is 3-nitrotyrosine. As with protein carbonyl formation, many studies have utilized anti-nitrotyrosine antibodies for immunoblotting of 2D gels before analysis of gel spots by MALDI peptide fingerprinting or untargeted MS/MS methods, and the same limitations apply. In this way, elevated levels of protein-bound 3-nitrotyrosine have been detected in proteins from brain tissue of Alzheimer’s disease patients [175] and in serum and colon during inflammatory bowel disease [176]. Using more rigorous MS approaches, sites of nitrotyrosine formation were identified on high density lipoprotein (HDL) and found to be increased during atherosclerosis [177]. Site-specific signatures of nitrotyrosine and chlorotyrosine in HDL by neutrophil extracellular trap enzymes have been observed in systemic lupus erythematosus (SLE) [178]. In human pituitary non-functional adenoma, nine nitro-proteins were identified using a nitrotyrosine affinity column (NTAC); the nitration sites were localized to functional domains of the proteins and it was suggested that might contribute to pathogenesis [120]. Interestingly, using MS-based strategies elevated levels of protein-bound 3-chlorotyrosine have been recently detected in mouse models of influenza [159], as well as in the clinical samples of inflammatory bowel disease [176], atherosclerosis [179], SLE [178] and post-myocardial infarction [180], providing evidence for the formation of chlorinating species in these inflammatory conditions.

## 7. Conclusions and Perspectives

Oxidative modifications of proteins and the regulation of signalling by oxPTMs are highly topical areas of increasingly recognized importance in biomedical science, and the increased levels of several oxPTMs in inflammatory diseases offer potential as biomarkers for the development of new diagnostics. It is clear that MS-based strategies have greatly underpinned the increase in knowledge in this area, and are confidently expected to continue to do so. The chemical enrichment and labelling approaches, together with the advanced MS/MS routines described, provide very powerful though time-consuming tools for investigating the relationships between specific oxidative modifications of proteins and mechanisms of disease pathology. There are many advances that are also helping to provide new information. MS imaging promises to be able to provide MS-based histology for mapping oxidative modifications across tissues, and although it has been used for mapping oxidised lipids [181], it has not yet been used to any significant degree for proteins. The availability, albeit at significant cost, of stable isotope-labelled animals (e.g., stable isotope labelling in mammals; SILAM) [182,183] may also provide a powerful tool for studying systemic or tissue specific oxidative stress and signalling. The use of genetic knockouts is a well-established method for unlocking cellular biochemical mechanisms and their roles in disease, and with the introduction of new technologies such as CRISPRi [184], it is set to become one of the key technologies for studies on pathology and for both mechanistic studies and validation. This has not yet been as widely exploited in the redox field as in others, or for studying redox biology in mammals as much as in plants, but promising results have been obtained from a range of studies (e.g., [185,186,187]). Kinetic and systems modelling has become well established in systems biology, and this is also now being applied to redox studies using MS and other data to build dynamic and predictive models that can help to understand the underlying biological processes complex regulatory dynamics of steady-state levels of protein oxidation [188].

However, there are still many challenges. It is essential to understand that analysis of oxPTMs involves non-standard proteomics methodology, and an important message of this review is that there are many potential pitfalls in the analysis of MS/MS data, which can lead to erroneous identifications of oxPTMs and conclusions. Consequently, it is essential to understand the requirements and limitations of the techniques used, and select appropriate approaches to address the research question. Although novel methods continue to be developed, their translation to early diagnosis tools for clinical settings continues to be difficult, owing to factors such as lack of well-established validation protocols for oxPTMs, the wide variety of methodologies, and complex data analysis [189]. In the meantime, the scientific community will continue to benefit from the advances in methodology and applications described in this article.

## Abbreviations

DNPH: 2,4-dinitrophenylhydrazine
DTT: dithiotreitol
ESI: electrospray ionization
HDL: high density lipoprotein
HPLC: high performance liquid chromatography
ICAT: isotope coded affinity tags
iTRAQ: isobaric tags for relative and absolute quantification
LC-MS or LC-MS/MS: Liquid chromatography coupled to mass spectrometry or tandem mass spectrometry
LPS: lipopolysaccharide
MALDI: matrix-assisted laser desorption/ionisation
MRM: multiple reaction monitoring
MS: mass spectrometry
MS/MS: tandem mass spectrometry
oxPTM: oxidative post-translational modification
PTM: post-translational modification
SLE: systemic lupus erythematosus
SNO: *S*-nitrosothiol
XIC: extracted ion chromatogram
